# Supplementary material for: Prime editing models the MTARC1 A165T variant in human liver organoids, demonstrating reduced steatosis, inflammation, and fibrosis
Source: Hepatol Commun. 2026 Sep 16;10(10):e1022. doi: 10.1097/HC9.0000000000001022 (PMC13585322; doi:10.1097/HC9.0000000000001022)
Supplement: Supplementary file 1 [file hc9-10-e1022-s001.docx]

**Supporting Information for**

Prime Editing Models the MTARC1 A165T Variant in Human Liver Organoids, Demonstrating Reduced Steatosis, Inflammation, and Fibrosis.

Amel Ben Saad^1^, Nahid Arghiani^1^, Arden D Weilheimer^1^, Stefan D Gentile^2^, Benjamin J Toles^1^, Sudipta Tripathi^3^, Seher Mohsin Sayed^3^, Anil Chandraker^3^, Alan C Mullen^1, 2, *^

* Corresponding author: Alan C Mullen

Email: alan.mullen@umassmed.edu

**This PDF file includes:**

Supplementary Figures 1 to 5

Tables S1 to S3

Supplementary Figures

Supplementary Figure 1. Sanger sequencing data for SNPs in *PNPLA3*, *TM6SF2*, *GCKR* and *HSD17B13* genes, showing that WA01 cells are heterozygous for *PNPLA3* rs738409 SNP, express *TM6SF2* and *GCKR* WT and carry the *HSD17B13* rs72613567 (TA insertion) SNP.

Supplementary Figure 2. MTARC1 expression was analyzed in HLOs that are homozygous for the WT genotype (G/G), the original hESC line that is heterozygous (G/A), and HLOs that are homozygous for the A165T genotype (A/A). HLOs were all differentiated in parallel. Analysis was performed on HLOs for lines 1 and 2 of the G/G and A/A genotypes and on two separate HLO differentiations from the orginal hESC line (G/A). Tubulin staining was performed as a loading control. Molecular weight markers are indicated on the left.

Supplementary Figure 3. Triglyceride levels and *THRB* mRNA expression in HLOs *MTARC1-WT* and *-A165T* under CTRL, PA and PA with resmetirom conditions.

Supplementary Figure 4. *CPT1A* mRNA expression quantified by RT-qPCR in HLOs *MTARC1-WT* and *-A165T* under CTRL, PA and PA with resmetirom conditions.

**Supplementary Figure 5**. *Vimentin* mRNA levels quantified by RT-qPCR. All 8 hESCs clones were differentiated at the same time. HLOs were harvested on day 21, and mRNA was extracted to check *Vimentin* levels. Data are representative of 3 independent experiments.

Tables

Table S1. Prime editing sequences.

|  | **Sequences (5' to 3')** |  |
| --- | --- | --- |
| epegRNA *MTARC1*-WT | GCTGGTTATCCACTGGGCGGGTTTTAGAGCTAGAAATAGCAAGTTAAAATAAGGCTAGTCCGTTATCAACTTGAAAAAGTGGCACCGAGTCGGTGCTGGCGAGGCCGCCGCCCAGTGGATATAATAATTCGCGGTTCTATCTAGTTACGCGTTAAACCAACTAGAA |  |
|  |  |  |
| epegRNA *MTARC1*-A165T | GCTGGTTATCCACTGGGCGGGTTTTAGAGCTAGAAATAGCAAGTTAAAATAAGGCTAGTCCGTTATCAACTTGAAAAAGTGGCACCGAGTCGGTGCTGGCGAGGCCACCGCCCAGTGGATATAATAATTCGCGGTTCTATCTAGTTACGCGTTAAACCAACTAGAA |  |
|  |  |  |
| Nicking sgRNA | GAAGTCACAGCCCTACCGCC |  |
| Primers | F: CAAAGCCATGTCTGCCCTTT |  |
|  | R: GGTAGGGCTGTGACTTCAGG |  |

Table S2. Primer sequences.

| **Genes** | **Sequences (5’ to 3’)** |
| --- | --- |
| *B-ACTIN* | F: CACAGAGCCTCGCCTTTGC |
|  | R: ATATCATCATCCATGGTGAGCTGG |
| *NANOG* | F: ACAGGTGAAGACCTGGTTCC |
|  | R: AAAGGCTGGGGTAGGTAGGT |
| *OCT4/POU5F1* | F: CTTGCTGCAGAAGTGGGTGGAGGAA |
|  | R: CTGCAGTGTGGGTTTCGGGCA |
| *MTARC1* | F: GCTTCCTGAAGTCACAGCCCTA |
|  | R: CAAGAATGGGCTGGTGTCTGAG |
| *COL1A1* | F: CAGGCTGGTGTGATGGGATT |
|  | R: AGCTCCAGCCTCTCCATCTT |
| *COL1A2* | F: TGGTCTCGGTGGGAACTTTG |
|  | R: CACCCTGTGGTCCAACAACT |
| *ACTA2* | F: CCAGAGCCATTGTCACACAC |
|  | R: CAGCCAAGCACTGTCAGG |
| *ACC1* | F: TTCACTCCACCTTGTCAGCGGA |
|  | R: GTCAGAGAAGCAGCCCATCACT |
| *FASN* | F: TTCTACGGCTCCACGCTCTTCC |
|  | R: GAAGAGTCTTCGTCAGCCAGGA |
| *CPT1A* | F: GATCCTGGACAATACCTCGGAG |
|  | R: CTCCACAGCATCAAGAGACTGC |
| *CPT2* | F: GCAGATGATGGTTGAGTGCTCC |
|  | R: AGATGCCGCAGAGCAAACAAGTG |
| *ACADAM* | F: AGAACCTGGAGCAGGCTCTGAT |
|  | R: GGATCTGGATCAGAACGTGCCA |
| *THRB* | F: GGTTGACTTGGAAGCCTTCAGC |
|  | R: GGATGATCTGGTCTTCACATGGC |

Table S3. List of antibodies used in the study.

| **Antibodies** | **Dilution** | **Vendor** | **Catalog number** | **Lot number** |
| --- | --- | --- | --- | --- |
| OCT3/4 | 1:100 | Santa Cruz | Sc-8628 | H2112 |
| NANOG | 1:100 | R and D Systems | AF1997 | KKJ0821091 |
| MTARC1 | 1:500 | Abcam | AB317262 | 1087594-4 |
| TUBULIN | 1:10000 | Proteintech | 66240-1-Ig | 10004491 |
| A-SMA | 1:1000 | Abcam | AB5694 | GR100205-1 |
| Collagen type I | 1:2000 | Proteintech | 14695-1-AP | 172128 |
| Alexa Fluor 488 | 1:500 | Thermo Scientific | A-11055 | 2411589 |
| Alexa Fluor 594 | 1:500 | Thermo Scientific | A-11058 | 1842799 |
| Goat anti-Rabbit IgG HRP | 1:1000 | Thermo Scientific | 32460 | 3220982 |
| Goat anti-Mouse IgG HRP | 1:1000 | Thermo Scientific | 31430 | 3212067 |
| DAPI | 1:10000 | Thermo Scientific | 62248 |  |
| BODIPY 493/503 | 0.5 ug/ml | Thermo Scientific | D3922 |  |
